# Incidental Brain Magnetic Resonance Imaging Findings and the Cognitive and Motor Performance in the Elderly: The Shanghai Changfeng Study

**DOI:** 10.3389/fnins.2021.631087

**Published:** 2021-02-19

**Authors:** Liangqi Wang, Huandong Lin, Yifeng Peng, Zehua Zhao, Lingyan Chen, Li Wu, Ting Liu, Jing Li, Anna Liu, Chun-Yi Zac Lo, Xin Gao

**Affiliations:** ^1^Department of Endocrinology and Metabolism, Zhongshan Hospital, Fudan University, Shanghai, China; ^2^Human Phenome Institute, Fudan University, Shanghai, China; ^3^School of Life Sciences, Fudan University, Shanghai, China; ^4^Department of Radiology, Putuo Hospital, Shanghai University of Traditional Chinese Medicine, Shanghai, China; ^5^Institute for Metabolic Diseases, Fudan University, Shanghai, China; ^6^Department of Geriatrics, Zhongshan Hospital, Fudan University, Shanghai, China; ^7^Institute of Science and Technology for Brain Inspired Intelligence, Fudan University, Shanghai, China

**Keywords:** magnetic resonance imaging, brain, incidental findings, aging, cognition

## Abstract

**Background:**

The frequently discovered incidental findings (IFs) from imaging observations are increasing. The IFs show the potential clues of structural abnormalities underlying cognitive decline in elders. Detecting brain IFs and their relationship with cognitive and behavioral functions helps provide the information for clinical strategies.

**Methods:**

Five hundred and seventy-nine participants were recruited in the Shanghai Changfeng Study. All participants performed the demographic, biochemical, and cognitive functions and gait speed assessment and underwent the high-resolution multimodal magnetic resonance imaging scans. We calculated the detection rate of brain IFs. The association between cardiovascular risk factors and IFs and the associations between IFs and cognitive and motor functions were assessed using regression models. The relationships among gray matter volume, cognitive function, and gait speed were assessed with/without adjusting the IFs to evaluate the effects of potential IFs confounders.

**Results:**

IFs were found in a total of 578 subjects with a detection rate of 99.8%. Age and blood pressure were the most significant cardiovascular risk factors correlated with IFs. IFs were found to be negatively associated with Montreal Cognitive Assessment, Mini-Mental State Examination, and gait speed. The gray matter volume was found to be positively correlated with the cognitive function without adjusting the white matter hyperintensity but not if adjusted.

**Conclusion:**

IFs are commonly found in the elderly population and related to brain functions. The adequate intervention of IFs related cardiovascular risk factors that may slow down the progression of brain function decline. We also suggest that IFs should be considered as confounding factors that may affect cognitive issues on the structural neuroimaging researches in aging or diseases.

## Introduction

Advanced magnetic resonance imaging (MRI) is widely used to study brain structures and functions (or activities) linking behaviors or cognitions in neuroscience (Westwood et al., 2014; Ikram et al., 2015; Hamer and Batty, 2019). The incidental findings (IFs) were characterized as unexpected imaging abnormalities with potential clinical significance in healthy research subjects or the findings that have no direct relationship to the research goal in patients recruited in the neuroimaging research (Illes et al., 2006). IFs can be classified into four categories: no referral necessary, routine referral, urgent referral, and immediate referral (Katzman and Patronas, 1999). IFs such as white matter hyperintensities (WMHs), enlarged perivascular space (EPVS), and cerebral microbleeds (CMBs) are commonly observed in the elderly population, and their prevalence is increasing with age (Liao et al., 1996; Longstreth et al., 1996; Ikram et al., 2008; Taki et al., 2011; Adams et al., 2015; Haller et al., 2018; Ramanoël et al., 2018). These IFs are also associated with potential clinical characteristics, which may increase the risk of adverse neurologic events, including stroke, mood problems, and dementia (Li et al., 2018). Most neuroimaging studies usually focus on neuroscientific topics such as healthy development, aging, or diseases. However, the frequently discovered IFs of the brain may increase the uncertainty of the main findings.

The detection of brain IFs is helpful to understand the natural course of diseases and guide clinical management (Vernooij et al., 2007). It is also helpful to ascertain the early sign for disease progression or development in an asymptomatic population, whereas the prevalence of neurological diseases differs by age, sex, and ethnic groups (Illes et al., 2004). Studying the proportion of these diseases in a specific population is crucial to avoid the cost–benefits and ethical issues strongly associated with the false-positive and false-negative rates (Illes et al., 2006). Previous neuroimaging researches have studied the brain structure and function in both healthy volunteers and patients, and there are IFs unexpectedly discovered by MRI. Katzman and Patronas (1999) reported a detection rate of 18% for unexpected brain abnormalities in a relatively young population whose mean age was 30.6 years from various magnetic resonance (MR) neuroimaging studies. A Rotterdam study found a high prevalence of meningiomas (0.9%) and aneurysms (1.8%) in the elderly population, and the presence of WMH and asymptomatic infarcts (7.2%) and the imaging features of the cerebral small vessel disease (CSVD) were increased with age (Vernooij et al., 2007). However, many studies did not include brain atrophy, EPVS, WMH, or CMBs as unexpected abnormalities, and the detection rates of IFs were between 1.74 and 15.6% (Yue et al., 1997; Tsushima et al., 2005; Weber and Knopf, 2006). Because the importance of these imaging markers might not be well understood in recent decades, these researches may underestimate the frequency due to the exclusion of these imaging markers.

The burden of dementia worldwide has increased rapidly over the past 30 years and continues to accelerate (Prince et al., 2015). WMH, CMBs, and brain atrophy were found to be correlated with cognitive decline, but the relationship between EPVS and cognitive performance remained inconsistent (Yao et al., 2014; Passiak et al., 2019; Wyss et al., 2019). Besides, previous studies claimed that CMBs were associated with gait disturbance independent of WMH and lacunar infarcts (de Laat et al., 2011b). The inconsistency of results leaving the relationship between these IFs and the cognitive and motor function remained unclarified. Appropriate treatments of cardiovascular risk factors such as lifestyle changes or use of medication might slow down the progression of these IFs and may further improve cognitive and motor function (Xiong et al., 2014). Therefore, identifying the risk factors in an asymptomatic population might provide an opportunity to prevent threatening symptoms from emerging. Furthermore, the IFs may influence the image processing, such as registration, segmentation, and parcellation, and impact the research result in the neuroimaging studies. WMH correlated to neuropsychiatric symptoms even at a subthreshold level. A previous study demonstrates that WMH is associated with subtle and subthreshold cognitive and neuropsychiatric symptoms (Spalletta et al., 2020). Depression patients present greater WMH volume even after controlling for cardiovascular confounders (Taylor et al., 2005). Thus, it is important to study the role of potential IFs such as WMH in the relationship between the MRI marker and the cognitive function. Our study aims to detect brain IFs, including brain atrophy, EPVS, WMH, and CMBs in a middle-aged and elderly Chinese population from Shanghai Changfeng study, based on the advanced MRI scanner and sequences, and to assess the risk factors of brain atrophy, EPVS, WMH, and CMBs, their relationship with cognitive and motor performance, as well as the potential IF confounders that may impact the neuroimaging study result.

## Materials and Methods

### Participants

The subjects were participants in the Shanghai Changfeng Study, a community-based residents study initiated in 2009, which adopted the research model used in the Rotterdam Study (Gao et al., 2010). From July 2017 to January 2020, we invited 718 subjects who had undergone the 5 year follow-up and had been older than 50 years to participate in the brain image acquisition. Eighty subjects refused to attend the MRI examination, 25 subjects had contraindications for MRI, five subjects encountered technical problems in the process of examination, 19 subjects could not complete the exam because of the long acquisition time, and 10 subjects had incomplete clinical data. Finally, 579 subjects (80.6%) finished the assessment of brain imaging in this population. The study was performed in accordance with the Declaration of Helsinki of 1975 and approved by the ethics committee of the Zhongshan Hospital, Fudan University, and each subject provided written informed consent.

### Image Acquisition

All images were acquired on a 3–T scanner (GE Discovery 750w, GE Healthcare) at Putuo Hospital, Shanghai. A 16-channel head–neck coil with 12 channels for the head was used. The high-resolution structural T1-weighted (T1w) MR images were acquired with a three-dimensional BRAVO sequence in the sagittal plane. Among the total participants, 80 subjects were scanned with a protocol, and others were scanned with another. The T1w imaging protocols for 80 participants were repetition time/echo time (TR/TE) = 8.6/3.2 ms, flip angle 14°, field of view (FOV) = 25.6, 148 slices, slice thickness 1 mm, matrix 256 × 256, and voxel size = 1 × 1 × 1 mm^3^; for 499 participants: TR/TE = 8.5/3.2 ms, flip angle 12°, FOV = 25.6, 172 slices, slice thickness 1 mm, matrix 256 × 256, and voxel size = 1 × 1 × 1 mm^3^; the T2-weighted (T2w) MR images were executed in the sagittal plane (TR/TE = 2,500/max ms, FOV = 25.6, 180 slices, slice thickness 1 mm, and matrix 256 × 256); the fluid-attenuated inversion recovery (FLAIR) sequence was acquired in the axial plane (TR/TE = 8,944/118.9 ms, flip angle 111°, FOV = 25.6, 51 slices, slice thickness 3 mm, and matrix 256 × 192); the susceptibility weighted imaging (SWI) sequence was performed in the axial plane (TR/TE = min/min ms, flip angle 12°, FOV = 25.0, 196 slices, slice thickness 1.6 mm, and matrix 320 × 224); and the phase-contrast MR angiography sequence was also obtained (TR/TE = min/min ms, flip angle 10°, FOV = 23.0, 40 slices, slice thickness 2 mm, and matrix 256 × 160). Proton density-weighted MR images were obtained instead of T2w MR images in 80 participants; the acquisition parameters were as follows: TR/TE = 10,000/8.1 ms, flip angle 111°, FOV = 25.0, 80 slices, slice thickness 2 mm, and matrix 416 × 256. The cushions were filled inside the coil to minimize motion artifacts generated during image acquisition.

### Assessment of Incidental Findings

All images were examined by two senior radiologists (L. Wang and Y. Tang) for unexpected abnormalities. Both of them were unaware of the clinical information. The MR images were reviewed on a PACS/DICOM software (OsiriX Lite, Geneva, Switzerland^1^), which allowed the multiplanar reconstruction. The IFs were classified into four categories: no referral necessary, routine referral, urgent referral, and immediate referral (Table 1; Katzman and Patronas, 1999). All diagnoses were based on imaging features with no further pathological confirmation. The definitions of each IFs are shown in Table 2.

**TABLE 1:** Classifications of incidental findings.

	Incidental findings
No referral necessary	WMH, brain atrophy, EPVS, CMBs, paranasal sinusitis, retention cysts of the maxillary sinus, mastoiditis, calvarial thickening
Routine referral	Pineal cyst, enlarged cisterna magna, asymptomatic brain infarct, empty sella, falx cerebri ossification, Rathke’s cleft cyst, hydrocephalus, cysts of the septum pellucidum, subdermal cyst
Urgent referral	Meningioma, pituitary adenoma, cavernous malformation, venous malformation, arachnoid cyst, osteoma, bone cyst, craniopharyngiomas, metastasis, chronic subdural hematoma
Immediate referral	Intracranial hemorrhage, i.e., acute subdural hematoma

*WMH, white matter hyperintensities; EPVS, enlarged perivascular space; CMBs, cerebral microbleeds.*

**TABLE 2:** Definitions of the incidental findings.

Imaging diagnosis	Definition based on MRI characteristics
White matter hyperintensities	WMH commonly locate in both periventricular or subcortical areas with hypointense on T1-weighted images and hyperintense on T2-weighted and FLAIR images.
Brain atrophy	Overall brain volume decreases with advancing age, and cerebral spinal fluid increases relatively.
Enlarged perivascular space	Enlarged perivascular space is also called Virchow–Robin space (VRS). VRS is commonly linear or lobulated in shape with hypointense on T1-weighted images and hyperintense on T2-weighted and FLAIR images.
Cerebral microbleeds	CMB is a hypointense foci on SWI images with maximum size up to 5 or even 10 mm.
Paranasal sinusitis	Unilateral or bilateral mucoperiosteal thickness or hydrops in the paranasal sinus.
Retention cysts of the maxillary sinus	Retention cysts of the maxillary sinus are rounded or dome-shaped lesions originating from the wall of the sinus with hypointense on T1-weighted images and hyperintense on T2-weighted image.
Calvarial thickening	Widened skull width.
Mastoiditis	Increased fluid signal in the mastoid air cells on T2-weighted images.
Pineal cyst	Cystic lesions in Pineal region with hypointense on T1-weighted images and hyperintense on T2-weighted images.
Asymptomatic brain infarct	Asymptomatic brain infarct is a focal parenchymal lesion with hypointense on T1-weighted images, hyperintense on T2-weighted images and with a hyperintense rim on FLAIR images.
Falx cerebri ossification	Falx cerebri ossification demonstrates signal intensity similar to fatty marrow on T1-weighted and T2-weighted images and hypointense on SWI images.
Empty Sella	Pituitary surface is concave, and the height of the pituitary is less than 3 mm.
Rathke’s cleft cyst	Rathke’s cleft cyst commonly locate between anterior and posterior pituitary lobes with hypointense on T1-weighted images and hyperintense on T2-weighted images.
Hydrocephalus	Ventricular enlargement out of proportion to sulcal atrophy.
Cysts of the septum pellucidum	A cystic structure between lateral ventricles whose walls are 10 mm apart or greater.
Subdermal cyst	Cystic lesions in subdermal with hypointense on T1-weighted images and hyperintense on T2-weighted images.
Enlarged cisterna magna	With more rostral extension in the posterior fossa close to the occipital squama than normal.
Venous malformation	Venous malformation is comprised of one or more atypically shaped veins and is hypointense on SWI images and/or flow-voids on T1-weighted and T2-weighted images.
Cavernous malformation	Cavernous malformations are usually hypointense on SWI images and with a hypointense hemosiderin rim and a core of variable signal intensity on T2-weighted images.
Meningioma	Meningioma is an extra-axial lesion with isointense or hypointense on T1-weighted images, variable signal intensities on T2-weighted images. Calcifications sometimes (hypointense on all images) within lesion.
Osteoma	Dense lesions usually involve outer table with hypointense on all images.
Arachnoid cyst	Arachnoid cyst is a well-defined cystic lesion with hypointense on T1-weighted images and hyperintense on T2-weighted images.
Bone cyst	Cystic lesion on skull with hypointense on T1-weighted images and hyperintense on T2-weighted images.
Craniopharyngiomas	A cystic or calcified tumor usually locate in suprasellar region with variable intensities.
Pituitary adenoma	Normal pituitary gland may not be identified. Signal intensities of pituitary adenoma are isointense to gray matter on all sequences but sometimes show cystic changes.
Metastasis	Multiple skull osteoclasia. Whole-body CT scan also showed multiple bone destruction surrounded by soft tissue masses.
Aneurysm	Aneurysms are well delineated focal arterial out-pouching with a saccular shape, usually located in cavernous internal carotid artery or circle of Willis. Aneurysms are visualized as flow voids (black) on T2-weighted images and could be well identified on magnetic resonance angiography.
Chronic subdural hematoma	Crescent-shaped fluid collection with hypointense on T1-weighted images and hyperintense on T2-weighted images. Extra-axial fluid collection could cross sutures.
Acute subdural hematoma	Crescent-shaped lesion with hyperintense on T1-weighted and T2-weighted images. Extra-axial lesion could cross sutures.

*WMH, white matter hyperintensities; EPVS, enlarged perivascular space; CMBs, cerebral microbleeds.*

### Image Processing

Among 579 participants, 515 participant’s images were qualified for post-processing. Because the sequence parameters of the T1w image were slightly different in 80 participants and the other 499 participants, we performed the image processing separately. The images acquired for each participant were reoriented to have the same spatial orientation, and parts of the neck below the cerebellum were removed using FSL (version: 5.0.11)^2^. Voxel-based morphometry analysis was conducted using SPM12 (version: 7484)^3^ in the MATLAB (R2018a) environment. The image was segmented into gray matter (GM), white matter (WM), and cerebral spinal fluid (CSF) with unified tissue segmentation and affinely registered to the standard Montreal Neurological Institute space (Ashburner and Friston, 2005). The diffeomorphic anatomical registration through exponentiated lie algebra (DARTEL) was used to refine the intersubject registration. The DARTEL initially computed a template based on the average tissue probability maps of all subjects and warped the GM and WM tissue maps of all subjects into the template. The images resulting from the first round were again used to create a second average template, to which images were again registered, and so on. The alignment of images and the template were increasingly good due to the iterative registration (Ashburner, 2007). In the last step of DARTEL, the GM tissues were modulated to preserve the amount of tissue in each voxel, and areas that were expanded during warping were correspondingly reduced in intensity (Good et al., 2001). Finally, the images were smoothed with a Gaussian kernel of 6 mm.

After preprocessing of the T1w image, the voxel size was 1.5 × 1.5 × 1.5 mm. We calculated the total GM volume (GMV), WM volume (WMV), and CSF volume by summing all voxels of certain brain tissue and yielded volumes in millimeters. Total intracranial volume (TIV) was the sum of GMV, WMV, and CSF volume.

For automatic segmentation of WMH on the FLAIR images, we used the lesion segmentation toolbox under the SPM12 with the lesion prediction algorithm lesion growth algorithm. The lesion segmentation toolbox provided the WMH volume and the WMH cluster numbers. The WMH cluster volume and WMH numbers were used to represent the severity of WMH. Subjects with WMH cluster number smaller than the number of 15 (the median of WMH cluster numbers) and WMH cluster volume smaller than the number of 3.21 ml (the median of WMH cluster volume) were defined as mild WMH, whereas those WMH cluster number equal or larger than 15 or WMH cluster volume equal or larger than 3.21 ml were defined as severe WMH (Yao et al., 2012).

Quantified GMV, WMV, TIV, WMH volume, and numbers acquired by the image processing method described earlier and dichotomous CMBs and EPVS, which were assessed based on MRI imaging features, were used in the subsequent analysis.

### Clinical Assessment

Information on demographics was obtained at baseline. Participants received a cognitive assessment with the Montreal Cognitive Assessment (MoCA) and the Mini-Mental State Examination (MMSE) 1 week before the MRI examination. Gait speed was recorded by a 6 m walking course, with an additional walking for 3 m at either end. Participants were instructed to walk to the end of the course at the usual speed, and assistive devices could be used if needed. The gait speed was tested for two walks. The mean speed of the two was used for analysis here. Body mass index was calculated as weight in kilograms divided by height in meters squared. We measured waist circumference in a standing position using a soft tape at the midpoint between the lowest rib and the iliac crest. Hip circumference was measured at the widest level over the greater trochanters. For blood pressure, the mean of three resting measurements was used for the analysis. The aspartate aminotransferase, alkaline phosphatase, and gamma-glutamyl transferase were measured using ultraviolet lactate and malate dehydrogenase methods. The serum creatinine and uric acid were measured using an autoanalyzer (Hitachi 7600; Hitachi, Tokyo, Japan) and standard methods. The fasting blood glucose (FBG) was measured using the glucose oxidase method. Hemoglobin A1c was also acquired using standard methods.

### Statistics

Continuous variables were described using the mean (standard deviation) and were compared using the Student’s *t*-test. Categorical variables were summarized as number (percent) and were compared using the chi-square test. We calculated the detection rate of all the brain IFs. If there were multiple lesions of the unexpected abnormalities, they would be considered as a single finding. For example, more than one CMBs or bilateral mastoiditis would be counted as one IF. We analyzed the age-specific distribution of the IFs by dividing the participants into three groups by age (53–59, 60–69, and 70–89 years). We also analyzed the distribution of the IFs by sex. A comparison of brain volume between with/without brain atrophy participants diagnosed by radiologists was performed for validation.

The association of cardiovascular risk factors and IFs (brain atrophy, EPVS, WMH, and CMBs) was assessed by binary logistic regression. Individuals with one of the following MRI markers, including WMH, brain atrophy, CMBs, and EPVS, were classified into the group with one CSVD marker. Individuals with two kinds of these markers were classified into the group with two CSVD markers. Individuals with three and four kinds of markers were grouped accordingly. The association of cardiovascular risk factors and CSVD groups was assessed by binary logistic regression, using individuals with 0–2 CSVD markers as the reference.

The associations of IFs (brain atrophy, EPVS, WMH, and CMBs) with log-transformed MMSE, log-transformed MoCA, and gait speed were assessed using multiple linear regression models. The multicollinearity of independent variables in the multivariate model was analyzed using the variance inflation factor (VIF). If the VIF is greater than 5, the multicollinearity is considered high (Xu et al., 2019). The associations of CSVD groups with log-transformed MMSE, log-transformed MoCA, and gait speed were performed using linear regression models.

We performed a linear regression among the GMV and log-transformed MMSE and log-transformed MoCA and gait speed. Owing to potential IF confounders, we created four adjustment models. The basic model did not adjust any covariates. Model 2 adjusted age, sex, and TIV. Model 3 further adjusted the severity of WMH, CMBs, and EPVS. Model 4 included age, sex, TIV, WMH volume, WMH cluster numbers, and CMBs and EPVS as covariates. We additionally examined the association between GMV and log-transformed MMSE, log-transformed MoCA, and gait speed both in the mild and severe WMH groups. *P*<0.05 was considered statistically significant. All statistical analyses were performed on MATLAB (version 2018a) and RStudio (version 1.2.5019) platform.

## Results

### Demographic Data

The basic characteristics of the participants are described in Table 3. The subjects included 240 males (41.45%) and 339 females (58.55%). The mean (SD) age was 67.58 (7.58) years, ranging from 53 to 89 years. There was evidence of differences between males and females in age, waist circumstance, hip circumstance, systolic blood pressure, diastolic blood pressure, gamma-glutamyl transferase, serum creatinine, uric acid, FBG, postprandial blood glucose, education, and MoCA score but not in body mass index, alanine aminotransferase, aspartate aminotransferase, alkaline phosphatase, blood urea nitrogen, hemoglobin A1c, gait speed, and MMSE score. See Table 3 and Supplementary Table 2 for detailed information. The sex differences of IFs are described in *Distribution of the incidental findings by age and sex*.

**TABLE 3:** Basic characteristics of participants of the Shanghai Changfeng study.

	Total	Males	Females	*P*
N	579	240	339	
Age (year)	67.58 (7.58)	68.90 (7.57)	66.65 (7.45)	<0.001
BMI (kg/m^2^)	24.75 (3.12)	25.02 (2.74)	24.56 (3.35)	0.080
Waist circumstance (cm)	82.63 (9.48)	86.57 (8.44)	79.83 (9.18)	<0.001
Hip circumstance (cm)	92.35 (5.91)	93.24 (4.86)	91.73 (6.49)	0.002
SBP (mmHg)	136.56 (20.20)	139.28 (19.97)	134.65 (20.17)	0.006
DBP (mmHg)	75.56 (10.05)	77.91 (10.72)	73.89 (9.21)	< 0.001
AST (U/L)	21.44 (9.48)	21.37 (8.76)	21.49 (9.97)	0.875
ALP (U/L)	71.07 (26.13)	69.16 (32.32)	72.42 (20.61)	0.140
CR (μmol/L)	73.92 (17.67)	85.75 (15.51)	65.54 (13.92)	< 0.001
UA (μmol/L)	318.78 (79.82)	357.75 (83.46)	291.19 (64.25)	< 0.001
FBG (mmol/L)	5.81 (1.33)	6.04 (1.53)	5.65 (1.15)	< 0.001
PBG (mmol/L)	7.89 (3.66)	8.27 (4.14)	7.62 (3.26)	0.035
HbA1c (%)	5.91 (0.87)	5.96 (1.03)	5.87 (0.74)	0.212
Education (years)	11.51 (3.23)	12.36 (3.40)	10.91 (2.97)	< 0.001
MMSE	29.09 (1.92)	29.22 (1.62)	28.99 (2.11)	0.157
MoCA	26.30 (2.76)	26.71 (2.38)	26.01 (2.96)	0.003
Gait speed (m/s)	1.11 (0.22)	1.12 (0.21)	1.11 (0.23)	0.619

*Mean (SD). AST, aspartate aminotransferase; ALP, alkaline phosphatase; CR, serum creatinine; UA, uric acid; FBG, fasting blood glucose; HbA1c, hemoglobin A1c*

### Detection Rate of Incidental Findings

**FIGURE 1:**
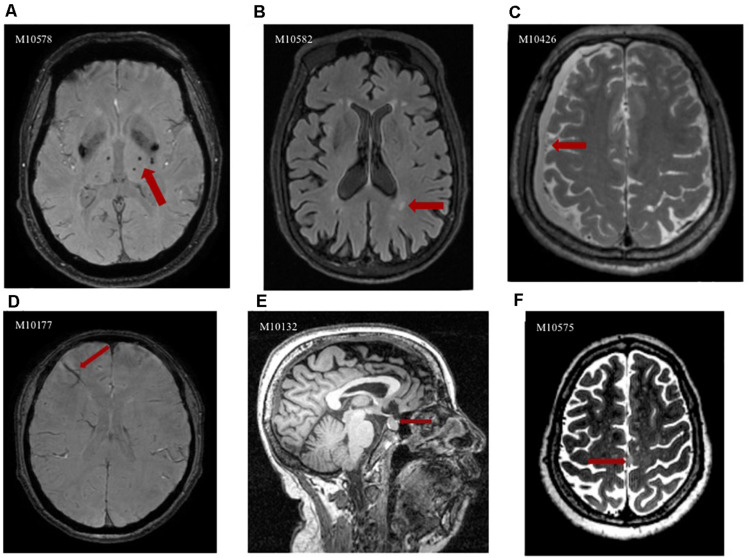
Abnormalities incidentally discovered in the Shanghai Changfeng study IFs could be observed on different images; here are some examples; the imaging features of the abnormalities are indicated with red arrows in each case. **(A)** Axial SWI image clearly depicts CMBs in the thalamus as hypointense areas; **(B)** Axial FLAIR image shows hyperintense areas in the deep white matter, representing WMH; **(C)** Axial T2-weighted image depicts crescent-shaped lesion that is isointense relative to the cerebrospinal fluid, findings that indicate chronic subdural hematoma; **(D)** Axial SWI image reveals venous malformation in the frontal lobe as hypointense atypically shaped areas; **(E)** Sagittal T1-weighted image demonstrates well-defined rounded foci that are isointense relative to gray matter in the sella turcica, representing pituitary adenoma; **(F)** Axial T2-weighted image shows meningioma as a hyperintense extra-axial mass that associated with the falx cerebri.

At least one abnormality was found in 578 subjects with a detection rate of 99.8%, according to the definitions described earlier. Table 4 shows the detection rate of each IF. Only one participant was found to be strictly normal without any IF, and none of the rest of the 578 participants reported any complaint. There were 99.7% of subjects with no referral necessary IFs, which WMHs (97.75%), brain atrophy (90.33%), EPVS (84.63%), and CMBs (39.03%) were the most frequent. The detection rates of routine referral, urgent referral, and immediate referral IFs were 29.2, 8.6, and 0%, respectively. The abnormalities in the pituitary region were of high prevalence among these two categories. We identified urgent referral IFs, including venous malformation in 14 participants (2.42%), a cavernous malformation in 11 participants (1.90%), arachnoid cyst in 8 participants (1.38%), and meningioma in 3 participants (0.52%). One case of craniopharyngiomas, two cases of chronic subdural hematoma, and one case of multiple skull metastasis were medically quite urgent. All diagnoses were based on imaging observations. We did not find a single case of an aneurysm, and no immediate referral cases were recorded. Figure 1 shows the abnormalities incidentally discovered in this study. The GMV and brain parenchyma volume (GMV + WMV) of the with-brain-atrophy group was significantly smaller than the without-brain-atrophy group, which confirmed the diagnosis of brain atrophy by the radiologists (Supplementary Table 3).

**TABLE 4:** Detection rate of each incidental finding.

	Incidental findings	
**No referral necessary**	***N* = 577 (99.7%)**	
	White matter hyperintensities	566(97.75%)
	Brain atrophy	523(90.33%)
	Enlarged perivascular space	490(84.63%)
	Cerebral microbleeds	226(39.03%)
	Paranasal sinusitis	196(33.85%)
	Retention cysts of the maxillary sinus	33(5.70%)
	Calvarial thickening	1(0.17%)
	Mastoiditis	13(2.25%)
**Routine referral**	***N* = 169 (29.2%)**	
	Pineal cyst	4(0.69%)
	Asymptomatic brain infarct	44(7.60%)
	Falx cerebri ossification	10(1.73%)
	Empty sella	55(9.50%)
	Rathke’s cleft cyst	52(8.98%)
	Hydrocephalus	1(0.17%)
	Cysts of the septum pellucidum	7(1.21%)
	Subdermal cyst	6(1.04%)
	Enlarged cisterna magna	11(1.90%)
**Urgent referral**	***N* = 50 (8.6%)**	
	Venous malformation	14(2.42%)
	Cavernous malformation	11(1.90%)
	Meningioma	3(0.52%)
	Osteoma	2(0.35%)
	Arachnoid cyst	8(1.38%)
	Bone cyst	2(0.35%)
	Craniopharyngiomas	1(0.17%)
	Pituitary adenoma	11(1.90%)
	Metastasis	1(0.17%)
	Chronic subdural hematoma	2(0.35%)

*In many cases, there was more than one finding in a person. All diagnoses were based on imaging observations.*

### Distribution of the Incidental Findings by Age and Sex

The brain atrophy, EPVS, CMBs, and asymptomatic infarct showed age-related increases in prevalence. The prevalence of brain atrophy, paranasal sinusitis, venous malformation, asymptomatic infarct, and falx cerebri ossification differed between males and females. The venous malformation and falx cerebri ossification were more prevalent in females than in males, whereas brain atrophy, paranasal sinusitis, and asymptomatic infarct were more prevalent in males than in females. A higher prevalence of multiple CSVD markers was observed in the elderly. However, no clear difference was found in the prevalence of multiple CSVD markers between males and females. Table 5 shows the distribution of IFs by age and sex. We did not show the abnormalities in less than 10 cases because the trend could not be observed clearly.

**TABLE 5:** Distribution of incidental findings by age and sex.

	Entire study (*N* = 579)	50–59 years (*N* = 89)	60–69 years (*N* = 263)	> 70 years (*N* = 227)	*P*-value (Chi-square)	Male (*N* = 240)	Female (*N* = 339)	*P*-value (Chi-square)
WMH	566(97.75%)	85(95.51%)	256(97.34%)	225(99.12%)	0.12	232(96.67%)	334(98.53%)	0.137
Brain atrophy	523(90.33%)	52(58.43%)	246(93.54%)	225(99.12%)	< 0.001*	231(96.25%)	292(86.14%)	< 0.001*
EPVS	490(84.63%)	67(75.28%)	216(82.13%)	207(91.19%)	< 0.001*	205(85.42%)	285(84.07%)	0.658
CMBs	226(39.03%)	25(28.09%)	98(37.26%)	103(45.37%)	0.013*	101(42.08%)	125(36.87%)	0.206
Paranasal sinusitis	196(33.85%)	25(28.09%)	93(35.36%)	78(34.36%)	0.446	99(41.25%)	97(28.61%)	0.002*
Cavernous malformation	11(1.90%)	1(1.12%)	3(1.14%)	7(3.08%)	0.246	4(1.67%)	10(2.95%)	0.322
Venous malformation	14(2.42%)	4(4.49%)	5(1.90%)	5(2.20%)	0.374	1(0.42%)	10(2.95%)	0.028*
Asymptomatic infarct	44(7.60%)	2(2.25%)	17(6.46%)	25(11.01%)	0.019*	36(15%)	8(2.36%)	< 0.001*
Empty sella	55(9.50%)	11(12.36%)	20(7.60%)	24(10.57%)	0.325	18(7.5%)	37(10.91%)	0.167
Rathke’s cleft cyst	52(8.98%)	7(7.87%)	29(11.03%)	16(7.05%)	0.284	23(9.58%)	29(8.55%)	0.67
Pituitary adenoma	11(1.90%)	2(2.25%)	5(1.90%)	4(1.76%)	0.960	2(0.83%)	9(2.65%)	0.114
Falx cerebri ossification	10(1.73%)	2(2.25%)	3(1.15%)	5(2.20%)	0.614	0(0%)	10(2.95%)	0.007*
Mastoiditis	13(2.25%)	3(3.37%)	4(1.52%)	6(2.64%)	0.52	7(2.92%)	6(1.77%)	0.359
Retention cysts of the maxillary sinus	33(5.70%)	6(6.74%)	14(5.32%)	13(5.73%)	0.883	16(6.67%)	17(5.01%)	0.398
**Number of CSVD markers**								
0	3(0.52%)	1(1.12%)	2(0.76%)	0(0%)	0.347	1(0.42%)	2(0.59%)	0.775
1	26(4.49%)	15(16.85%)	10(3.8%)	1(4.41%)	< 0.001*	7(2.92%)	19(5.6%)	0.124
2	73(12.61%)	23(25.84%)	35(13.31%)	15(6.61%)	< 0.001*	26(1.3%)	47(13.86%)	0.279
3	275(47.5%)	32(35.96%)	128(48.67%)	115(50.66%)	0.055	114(47.5%)	161(47.49%)	0.997
4	202(34.89%)	18(20.22%)	88(33.46%)	96(42.29%)	< 0.001*	92(38.33%)	110(32.45%)	0.143

N (%). We did not show the abnormalities in less than 10 cases because the trend could not be observed clearly. *p < 0.05.

### Correlations Between Cardiovascular Risk Factors and Incidental Findings

**FIGURE 2:**
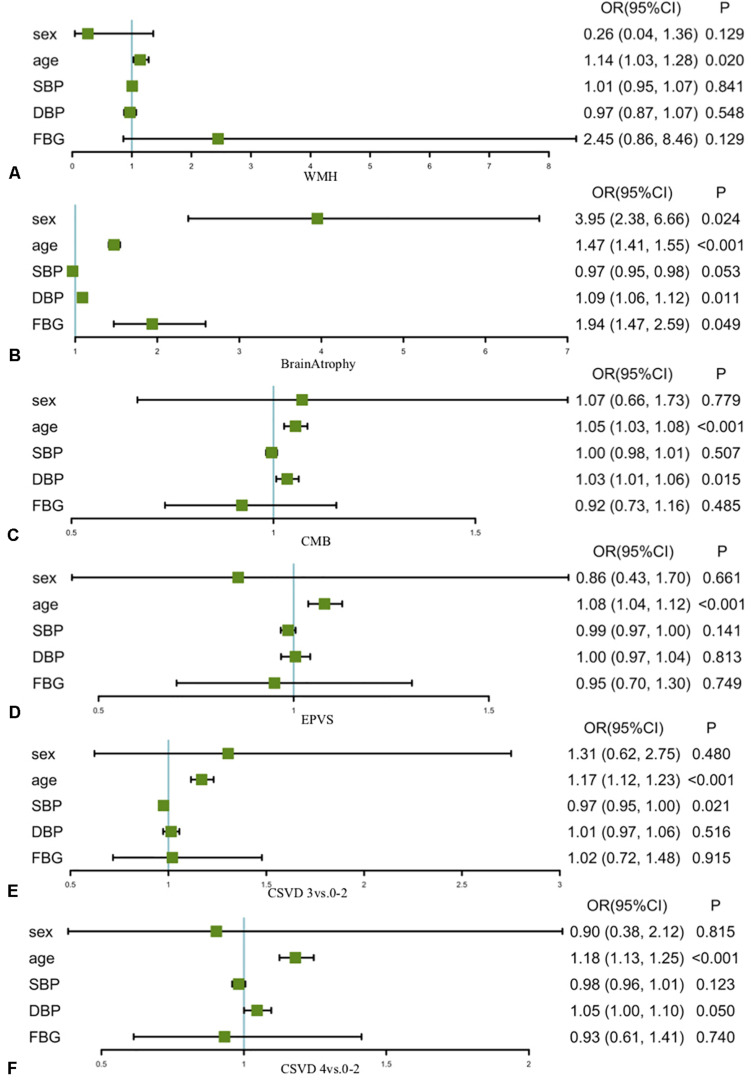
Correlations between cardiovascular risk factors and incidental findings Associations of clinical characteristics with the incidental findings among the participants of the Shanghai Changfeng Study. Age was found to be correlated with **(A)** WMH, **(B)** brain atrophy, **(C)** CMBs, and **(D)** EPVS and was associated with increasing severity of CSVD markers (**E**, CSVD 3 vs. 0–2; **F**, CSVD 4 vs. 0–2). DBP correlated with brain atrophy and CMBs. FBG was associated with brain atrophy, and males were more likely to develop brain atrophy. OR, odds ratio (95% confidence interval). WMH, white matter hyperintensities; CMBs, cerebral microbleeds; EPVS, enlarged perivascular space; CSVD, cerebral small vessel disease; SBP, systolic blood pressure; DBP, diastolic blood pressure; FBG, fasting blood glucose; PBG, postprandial blood glucose; BMI, body mass index.

Age and blood pressure were found to be the most significant cardiovascular risk factor correlated with IFs (Figure 2 and Supplementary Table 1). Per year increase in age, the risks of WMH, brain atrophy, CMBs, and EPVS increased by 14, 47, 8, and 5%, respectively. The risks of CMBs and brain atrophy increased by 3 and 9% per millimeters of mercury increase in diastolic blood pressure, respectively. Age was found to be associated with increasing severity of CSVD markers, and the magnitude of the association slightly increased with the severity of CSVD markers (odds ratios increased from 1.17 to 1.18). In addition, FBG was associated with brain atrophy, and males were more likely to develop brain atrophy.

### Relationship Between Incidental Findings and Cognitive Function as Well as Gait Speed

The VIFs of WMH, brain atrophy, EPVS, and CMBs were 1.06, 1.10, 1.12, and 1.05, respectively, indicating no apparent multicollinearity (<5) in the model. EPVS was found to be negatively associated with MoCA score, whereas brain atrophy and CMBs were negatively associated with gait speed (Table 6). The severity of CSVD markers was found to be related to gait speed.

**TABLE 6:** Relationship between incidental findings and cognitive function as well as gait speed.

	MMSE		MoCA		Gait speed	
CSVD markers	β (SD)	*P*	β (SD)	*P*	β (SD)	*P*
WMH	−0.03 (0.04)	0.442	−0.05 (0.05)	0.327	−0.06 (0.06)	0.308
Brain atrophy	−0.01 (0.02)	0.528	0.01 (0.03)	0.781	−**0.08 (0.03)**	**0.010**
EPVS	−0.02 (0.01)	0.136	−**0.04 (0.01)**	**0.018**	−0.02 (0.02)	0.254
CMBs	0.02 (0.02)	0.185	0.00 (0.02)	0.825	−**0.13 (0.03)**	**< 0.001**
**Number of CSVD markers**						
3 vs. 0–2	**0.02 (0.01)**	**< 0.001**	0.02 (0.01)	0.113	−**0.16 (0.02)**	**< 0.001**
4vs. 0–2	−0.01 (0.02)	0.809	−0.03 (0.03)	0.325	−**0.17 (0.03)**	**< 0.001**

*Words in bold indicate the significant correlation. CSVD, cerebral small vessel disease; WMH, white matter hyperintensities; EPVS, enlarged perivascular space; CMBs, cerebral microbleeds.*

The GMV was found to be positively correlated with cognitive function and gait speed without regressing WMH. However, the relationship became insignificant after adjusting the WMH volume and WMH cluster numbers (Table 7). The same relationship was observed in the severe WMH group, in which the GMV was found to be correlated to cognitive function, but the correlation disappeared after adjusting the WMH volume and WMH cluster numbers.

**TABLE 7:** Relationship between GMV and cognitive function as well as gait speed.

	MMSE		MoCA		Gait speed	
GMV (per 1,000 ml)	β (SD)	*P*	β (SD)	*P*	β (SD)	*P*
**Whole population (*N* = 515)**						
Model 1	**0.33 (0.11)**	**0.003**	**0.58 (0.12)**	**< 0.001**	**0.75 (0.16)**	**< 0.001**
Model 2	**0.35 (0.14)**	**0.016**	**0.49 (0.16)**	**0.002**	0.38 (0.20)	0.057
Model 3	**0.32 (0.15)**	**0.027**	**0.44 (0.16)**	**0.005**	0.35 (0.20)	0.080
Model 4	0.28 (0.17)	0.098	0.34 (0.19)	0.070	0.39 (0.23)	0.095
**Severe WMH group (*N* = 324)**						
Model 1	**0.47 (0.17)**	**0.005**	**0.76 (0.17)**	**< 0.001**	**0.83 (0.19)**	**< 0.001**
Model 2	**0.50 (0.21)**	**0.020**	**0.66 (0.22)**	**0.003**	0.40 (0.23)	0.085
Model 4	0.44 (0.27)	0.100	0.48 (0.28)	0.085	0.42 (0.29)	0.142
**Mild WMH group (*N* = 191)**						
Model 1	−0.09 (0.07)	0.196	0.07 (0.13)	0.581	0.22 (0.32)	0.490
Model 2	−0.03 (0.09)	0.703	0.05 (0.17)	0.778	0.64 (0.41)	0.117
Model 4	−0.03 (0.09)	0.723	0.05 (0.17)	0.759	0.46 (0.40)	0.260

*Words in bold indicate the significant correlation. GMV, gray matter volume; CMB, cerebral microbleeds; EPVS, enlarged perivascular space. Linear regression between the GMV and log-transformed MMSE, log-transformed MoCA and gait speed. Model 1 did not adjust any covariates. Model 2 adjusted age, sex, and TIV. Model 3 further adjusted the severity of WMH, CMBs, and EPVS. Model 4 included age, sex, TIV, WMH volume, WMH cluster numbers, CMBs, and EPVS as covariates. We additionally examined the association between GMV and log-transformed MMSE, log-transformed MoCA and gait speed both in the mild WMH group and the severe WMH group.*

## Discussion

The present study identified IFs from brain MRI in a cohort of Chinese subjects aged 53–89 years. Most of the participants presented unexpected brain abnormalities, showing WMH, brain atrophy, EPVS, and CMBs. Among routine referral and urgent referral categories, the abnormalities in the pituitary region and the vascular malformation were the most frequent. The brain atrophy, EPVS, and CMBs showed age-related increases in prevalence. Age and blood pressure were found to be the major risk factors associated with IFs. EPVS was found to be negatively correlated with cognitive function, whereas brain atrophy and CMBs were negatively associated with gait speed. The GMV was found to be positively correlated with the cognitive function, but the significance disappeared after adjusting the WMH volume and WMH cluster numbers. With improved high-resolution images, quantified methods, and a larger cohort, our result providing the detection rate of IFs may help guide the clinical management and understand the natural course of diseases. Our results indicate that potential IFs showed the inference for the neuroscientific analysis, and the IF confounders could be helpful for future neuroimaging studies.

WMH was the most frequent unexpected finding in the no-referral-necessary category. Brain atrophy, EPVS, and CMBs were commonly observed among findings besides WMH in that category. As expected, the detection rates of these unexpected abnormalities were increased with advancing age. Many pieces of research have shown evidence that brain atrophy, EPVS, WMH, and CMBs are correlated with age (Liao et al., 1996; Longstreth et al., 1996; Ikram et al., 2008; Taki et al., 2011; Adams et al., 2015; Haller et al., 2018; Ramanoël et al., 2018). Similar to our study, WMH was also found to be significantly increased with age (Vernooij et al., 2007). The prevalence of WMH was 94.6% in 45–59 years and increased to 98% in participants older than 75 years old. We found 519 (90.26%) of 579 participants with brain atrophy, which was close to the prevalence (99%) in a population older than 65 years (Manolio et al., 1993). In a Chinese ischemic stroke population study, the prevalence rates of EPVS and CMBs were 100 and 23.3%, respectively (Zhang, 2015). The percentage of CMBs (38.96%) in our study was considerably higher owing to the use of SWI. SWI sequence enables better depiction of CMBs in comparison with T2^∗^-weighted gradient echo sequence. Approximately twice as many CMBs were suggested on SWI compared with T2^∗^-weighted gradient echo imaging (Goos et al., 2011). Brain atrophy, WMH, EPVS, and CMBs are associated with stroke, progressive cognitive decline, dementia, gait abnormality, and psychological problems (van der Flier et al., 2005; de Laat et al., 2011a; Xu et al., 2017). Early information on these IFs would be helpful to aid clinical management.

Among routine and urgent referral categories, the detection rates of pituitary lesions were higher than the prevalence reported in previous researches. There were 1.91% of participants with pituitary adenoma in our study, whereas Tsushima et al. (2005) concluded a frequency of less than 0.1% in a population aged 22–84 years. The discovery of pituitary adenoma ranged from 3 to 27% at autopsy (Buurman and Saeger, 2006). Although the subtle abnormalities might still remain unobservable as compared with autopsy studies, the detection rate of pituitary adenoma was higher in this high-resolution study than in previous imaging researches. Autopsy research demonstrated that the percentage of Rathke’s cleft cyst was 11.3% in a group of older than 30 years (Teramoto et al., 1994). Rathke’s cleft cyst was confirmed in 8.87% of participants in our study, which was within an acceptable range. We identified 9.39% of participants with empty sella syndrome, but Weber and Knopf (2006) only reported 0.35% in a younger age group. Smaller lesions in the pituitary region could be discovered because we used the sagittal plane acquisition. The high-resolution images, which allowed coronal plane reconstruction, increased our sensitivity to track these lesions as well.

Venous malformation (2.26%) and cavernous malformation (1.91%) were highly prevalent in our study than in previous studies. The prevalence of cavernous malformation was 0.4% in the Rotterdam study (Vernooij et al., 2007). Tsushima et al. (2005) discovered that the venous malformation was less than 0.1% in their adult population. The higher discovery rate in this study may be due to the SWI, which is more sensitive to lesion detection. We found no intracranial aneurysms, although the images had been carefully read. Because we did not perform the contrast-enhanced imaging and the MR angiography imaging with the Willis cycle, smaller aneurysms may be overlooked in the routine T1w and T2w images.

Aging and blood pressure were found to be the major risk factors associated with brain atrophy, EPVS, WMH, and CMBs. These findings were consistent with previous work showing that these IFs were related to the growing age and hypertension (Hilal et al., 2017). Our results also suggested that aging and hypertension contributed to multiple imaging markers. It is plausible that among elderly individuals free of clinical manifestations such as the current cohort, IFs may coexist and share similar risk factors. Moreover, increasing age and blood pressure were associated with a severe burden of IFs, indicating that aging and hypertension might be the factors responsible for the prevalence and severity of the IFs.

Aged people with EPVS had a higher likelihood of poor cognitive performance. A previous study demonstrated that EPVS might have critical relevance with cognitive function, especially information processing speed and executive function (Passiak et al., 2019). However, no significant association between EPVS and cognitive performance was observed in another study (Yao et al., 2014). Our result was suggestive of the association between EPVS and cognitive function. Unique from other IFs, EPVS reflects fluid extravasation from increased intraluminal pressure and pulsatility. The association of EPVS with cognition in the current study highlight that in elderly individuals, EPVS may hold higher cognitive relevance than WMH, EPVS, and brain atrophy through distinct vessel pathology. The result in the current study also emphasizes the importance that the EPVS should be reported in routine neuroimaging reports.

Brain atrophy and CMBs were found to be negatively correlated to gait speed. A previous study showed that CMBs might be associated with gait disturbance, characterized as lower gait velocity, independent of WMH and lacunar infarcts (de Laat et al., 2011b). In line with this study, results presented here suggested that the CMBs were correlated with gait speed but coexisted with brain atrophy. Our result also showed that participants with a severe burden of IFs, represented by the coexistence of multiple imaging markers, were associated with slower gait speed. Different IFs might have unique pathways that contributed to clinical manifestation based on separate pathologies, but the coexistence of IFs may indicate the early disturbance of lower extremity motor function in this population free of clinical manifestation.

The GMV was found to be associated with the cognitive function, but the significance was disappeared after adjusting the WMH in the current study. Because the coexistence of WMH, CMBs, EPVS, and brain atrophy was of high prevalence in this elderly population, it was unconfirmed whether the relationship between GMV and cognition was influenced by potential IF confounders. Numerous studies demonstrate that a correlation exists between brain volume and cognitive function (Ikram et al., 2010; Debette et al., 2011). However, the interference of IFs, such as WMH volume on GMV, is still a matter of debate. We extended the findings by controlling the potential IF confounders and clarifying the relationship between GMV and cognitive function. The result highlighted the pivotal role of WMH in the relationship between GMV and the cognitive function, suggesting that future studies of GMV should consider the impact of IFs such as the WMH, especially in the elderly population.

The strength of our study was that our research was a relatively large sample size study conducted in an elderly community-based cohort. We also minimized the detection bias as small as possible that the radiologists were unaware of the clinical history. The high-resolution images provided the advantages for detecting subtle abnormalities and the FLAIR and SWI enhanced the depiction of WMH and CMBs as well. Therefore, the advances in technology helped improve the detection of IFs in our study, which indicated that the detection rate depended markedly on the scanner and sequence characteristics. However, there are some limitations to the current study. Our subjects were elderly and middle-class Chinese. The results of our homogeneous population might not be extendable to other demographic or ethnic groups. There was no histological confirmation of any unexpected abnormalities, but the imaging diagnoses listed in Table 2 were generally typical. The prevalence might be reduced because we did not include the contrast-enhanced imaging, especially for the detection of the aneurysm. Finally, a small proportion of our study underwent proton density-weighted imaging instead of T2w imaging, making the detection rate slightly different.

In conclusion, IFs are commonly found in the elderly population, indicating that people with normal cognitive and clinical manifestations could have underlying structural changes in the brain. The participants will be followed up in the future to clarify the outcome of IFs. High-resolution brain imaging is very valuable for the early detection of small changes in older people. The adequate intervention of IFs related cardiovascular risk factors may further slowdown the progression of the cognitive and motor function decline. Furthermore, IFs such as WMH should be considered as a factor that may affect cognitive issues on the structural neuroimaging researches in aging or diseases. Therefore, the detection rate of IFs may help guide the clinical management and understand the natural course of diseases, and our results indicating potential IF confounders could be helpful for the future neuroimaging study design.

## Data Availability Statement

The raw data supporting the conclusions of this article will be made available by the authors, without undue reservation.

## Ethics Statement

The studies involving human participants were reviewed and approved by the ethics committee of the Zhongshan Hospital, Fudan University. The patients/participants provided their written informed consent to participate in this study.

## Author Contributions

XG and C-YL designed the research. HL, LC, LW, TL, and JL performed the ChangFeng Study to collect clinical and neuropsychological data. LW, YP, ZZ, and AL collected the neuroimaging data. C-YL and LW analyzed the data. LW wrote the manuscript. XG and C-YL revised the manuscript. All authors contributed to the article and approved the submitted version.

## Conflict of Interest

The authors declare that the research was conducted in the absence of any commercial or financial relationships that could be construed as a potential conflict of interest.

## Footnotes

1 https://www.osirix-viewer.com/osirix/osirix-md/download-osirix-lite/

2 https://fsl.fmrib.ox.ac.uk/fsl/fslwiki/FslInstallation

3 https://www.fil.ion.ucl.ac.uk/spm/
