# Supplementary material for: Incidental Brain Magnetic Resonance Imaging Findings and the Cognitive and Motor Performance in the Elderly: The Shanghai Changfeng Study
Source: Front Neurosci. 2021 Feb 19;15:631087. doi: 10.3389/fnins.2021.631087 (PMC7933572; doi:10.3389/fnins.2021.631087)
Supplement: Supplementary file 1 [file Table_1.docx]

**Supplementary Materials**

Table S1: Correlations between cardiovascular risk factors and IFs

| **CSVD markers** | **sex** | **age** | **BMI** | **Waist** | **HIP** | **Educa-tion** | **SBP** | **DBP** | **ALP** | **AST** | **GGT** | **CR** | **UA** | **FBG** | **PBG** | **HbA1c** |
| --- | --- | --- | --- | --- | --- | --- | --- | --- | --- | --- | --- | --- | --- | --- | --- | --- |
| **WMH** | 0.26  (0.04, 1.36) | **1.14**  **(1.03, 1.28) *** | 1.08  (0.72, 1.61) | 0.98  (0.90, 1.13) | 1.08  (0.92, 1.21) | 0.89  (0.71, 1.11) | 1.01  (0.95, 1.07) | 0.97  (0.87, 1.07) | 0.99  (0.95, 1.02) | 1.09  (0.97, 1.30) | 1.08  (1.01, 1.20) | 1.00  (0.95, 1.07) | 1.00  (0.99, 1.01) | 2.45  (0.86, 8.46) | 0.94  (0.71, 1.28) | 1.58  (0.34, 10.14) |
| **Brain atrophy** | **3.95**  **(2.38, 6.66) *** | **1.47**  **(1.41, 1.55) ***** | 1.08  (0.98, 1.20) | 0.95  (0.92, 0.98) | 0.97  (0.93, 1.01) | 1.04  (0.98, 1.11) | 0.97  (0.95, 0.98) | **1.09**  **(1.06, 1.12) *** | 0.99  (0.98, 1.00) | 0.98  (0.97, 1.00) | 1.01  (1.00, 1.03) | 1.00  (0.99, 1.01) | 1.00  (0.99, 1.00) | **1.94**  **(1.47, 2.59) *** | 1.00  (0.93, 1.06) | 1.00  (0.71, 1.42) |
| **EPVS** | 0.86  (0.43, 1.70) | **1.08**  **(1.04, 1.12) ***** | 1.08  (0.93, 1.26) | 0.96  (0.92, 1.00) | 1.02  (0.96, 1.08) | 1.04  (0.96, 1.12) | 0.99  (0.97, 1.00) | 1.00  (0.97, 1.04) | 1.00  (0.99, 1.01) | 1.00  (0.97, 1.03) | 1.00  (0.99, 1.00) | 1.01  (0.99, 1.03) | 1.00  (1.00, 1.01) | 0.95  (0.70, 1.30) | 1.07  (0.99, 1.16) | 1.03  (0.64, 1.71) |
| **CMBs** | 1.07  (0.66, 1.73) | **1.05**  **(1.03, 1.08) ***** | 0.97  (0.87, 1.08) | 0.99  (0.96, 1.03) | 0.98  (0.94, 1.02) | 1.01  (0.96, 1.07) | 1.00  (0.98, 1.01) | **1.03**  **(1.01, 1.06) *** | 1.00  (0.99, 1.01) | 1.01  (0.99, 1.03) | 1.00  (0.99, 1.00) | 1.00  (0.99, 1.02) | 1.00  (1.00, 1.00) | 0.92  (0.73, 1.16) | 1.02  (0.96, 1.08) | 1.14  (0.81, 1.64) |
| **Number of CSVD markers** | | | | | | | | | | | | | | | | |
| **3 vs. 0-2** | 1.31  (0.62, 2.75) | **1.17**  **(1.12, 1.23) ***** | 1.10  (0.93, 1.30) | 0.98  (0.93, 1.02) | 1.00  (0.93, 1.07) | 1.06  (0.97, 1.16) | **0.97**  **(0.95, 1.00) *** | 1.01  (0.97, 1.06) | 1.00  (0.98, 1.01) | 1.01  (0.98, 1.05) | 1.00  (0.99, 1.01) | 1.00  (0.98, 1.02) | 1.00  (1.00, 1.01) | 1.02  (0.72, 1.48) | 1.05  (0.96, 1.15) | 1.31  (0.75, 2.35) |
| **4 vs. 0-2** | 0.90  (0.38, 2.12) | **1.18**  **(1.13, 1.25) ***** | 1.05  (0.88, 1.27) | 0.96  (0.91, 1.02) | 0.98  (0.91, 1.06) | 1.05  (0.96, 1.16) | 0.98  (0.96, 1.01) | 1.05  (1.00, 1.10) | 1.00  (0.98, 1.01) | 1.01  (0.98, 1.06) | 1.00  (0.99, 1.00) | 1.01  (0.99, 1.03) | 1.00  (1.00, 1.00) | 0.93  (0.61, 1.41) | 1.11  (1.00, 1.23) | 1.20  (0.62, 2.40) |

OR (95% confidence interval). WMH, white matter hyperintensities; EPVS, enlarged perivascular space; CMBs, cerebral microbleeds.

Table S2：abbreviations

| **abbreviations** | | **Full title** |
| --- | --- | --- |
| ALP | alkaline phosphatase | |
| ALT | alanine aminotransferase | |
| AST | aspartate aminotransferase | |
| BMI | body mass index | |
| BUN | urea nitrogen | |
| CMBs | cerebral microbleeds | |
| CR | serum creatinine | |
| CSVD | cerebral small vessel disease | |
| DBP | diastolic blood pressure | |
| EPVS | enlarged perivascular space | |
| FBG | fasting blood glucose | |
| FLAIR | fluid-attenuated inversion recovery | |
| GGT | gamma-glutamyl Transferase | |
| GMV | gray matter volume | |
| HbA1c | hemoglobin A1c | |
| HDL-c | HDL-cholesterol | |
| LDL-c | LDL-cholesterol | |
| MMSE | Mini-Mental State Examination | |
| MoCA | Montreal Cognitive Assessment | |
| MRI | magnetic resonance imaging | |
| PBG | post-load blood glucose | |
| PC MRA | phase contrast magnetic resonance angiography | |
| SBP | systolic blood pressure | |
| SWI | susceptibility weighted imaging | |
| TC | total cholesterol | |
| TG | triacylglycerol | |
| TIV | total intracranial volume | |
| UA | uric acid | |
| VBM | voxel-based morphometry | |
| VIF | variance inflation factor | |
| WHR | waist-to-hip ratio | |
| WMH | white matter hyperintensities | |
| WMV | white matter volume | |

Table S3: Group comparison between brain atrophy and normal participants

|  | Brain atrophy | Normal | P value |
| --- | --- | --- | --- |
| N | 465 | 50 |  |
| GMV | 601.11 (62.77) | 638.53 (51.78) | <0.001 |
| WMV | 406.64 (48.40) | 416.94 (44.03) | 0.125 |
| GMV+WMV | 1007.75 (98.37) | 1055.47 (87.74) | 0.001 |

Mean (SD). N: number of participants; GMV: gray matter volume; WMV: white matter volume.
